# Contiguous 22.1-kb deletion embracing *AVPR2* and *ARHGAP4* genes at novel breakpoints leads to nephrogenic diabetes insipidus in a Chinese pedigree

**DOI:** 10.1186/s12882-018-0825-5

**Published:** 2018-02-02

**Authors:** Ying Bai, Yibing Chen, Xiangdong Kong

**Affiliations:** Genetics and Prenatal Diagnosis Center, The First Affiliated Hospital of Zhengzhou University, 1 Jianshe Road East, Zhengzhou, Henan 450052 China

**Keywords:** Nephrogenic diabetes insipidus, *AVPR2*, Deletion, Genetic diagnosis

## Abstract

**Background:**

It has been reported that mutations in arginine vasopressin type 2 receptor (*AVPR2)* cause congenital X-linked nephrogenic diabetes insipidus (NDI). However, only a few cases of *AVPR2* deletion have been documented in China.

**Methods:**

An NDI pedigree was included in this study, including the proband and his mother. All NDI patients had polyuria, polydipsia, and growth retardation. PCR mapping, long range PCR and sanger sequencing were used to identify genetic causes of NDI.

**Results:**

A novel 22,110 bp deletion comprising *AVPR2* and *ARH4GAP4* genes was identified by PCR mapping, long range PCR and sanger sequencing. The deletion happened perhaps due to the 4-bp homologous sequence (TTTT) at the junctions of both 5′ and 3′ breakpoints. The gross deletion co-segregates with NDI. After analyzing available data of putative clinical signs of *AVPR2* and *ARH4GAP4* deletion, we reconsider the potential role of *AVPR2* deletion in short stature.

**Conclusions:**

We identified a novel 22.1-kb deletion leading to X-linked NDI in a Chinese pedigree, which would increase the current knowledge in *AVPR2* mutation.

## Background

Nephrogenic diabetes insipidus (NDI) is a group of diseases characterized by inability to concentrate urine in response to arginine vasopressin (AVP) [1]. The main clinical manifestation of NDI is polyuria and/or polydipsia. X-linked NDI, caused by genetic defect in the arginine vasopressin V2 receptor (AVPR2), accounts for 90% of NDI cases. The remaining 10% of the NDI cases are mainly related to *AQP2* gene mutations [2], which is autosomally inherited. For X-linked NDI, males with pathogenic mutations in *AVPR2* are affected, while heterozygous females show various degrees of penetrance [3]. Furthermore, skewed X inactivation, which is preferential methylation of the normal allele of the *AVPR2* gene, can cause NDI in female heterozygotes [4].

*AVPR2* is located on Xq28 and centromeric to the adjacent *ARHGAP4* gene that encodes rho GTPase activating protein 4. *AVPR2* consists of three exons and encodes a 371-amino acid G protein-coupled receptor. To date, more than 277 *AVPR2* putative disease-causing mutations have been reported (http://www.hgmd.cf.ac.uk/ac/index.php). Large deletions that lead to complete loss of *AVPR2* and parts of the neighboring genes *ARHGAP4* or L1 cell adhesion molecule (*L1CAM*) have also been reported [3, 5–12]. However, very few X-linked NDI cases caused by gross *AVPR2* deletion have been reported in China.

In this study, we identified a novel gross deletion covering entire *AVPR2* locus and approximately half of *ARHGAP4* in a Chinese pedigree with NDI. In order to distinguish the putative clinical signs of an *AVPR2* deletion, we reviewed all characterized *AVPR2* deletions and found the potential role of *AVPR2* in short stature.

## Methods

### Patients

**Fig. 1:**
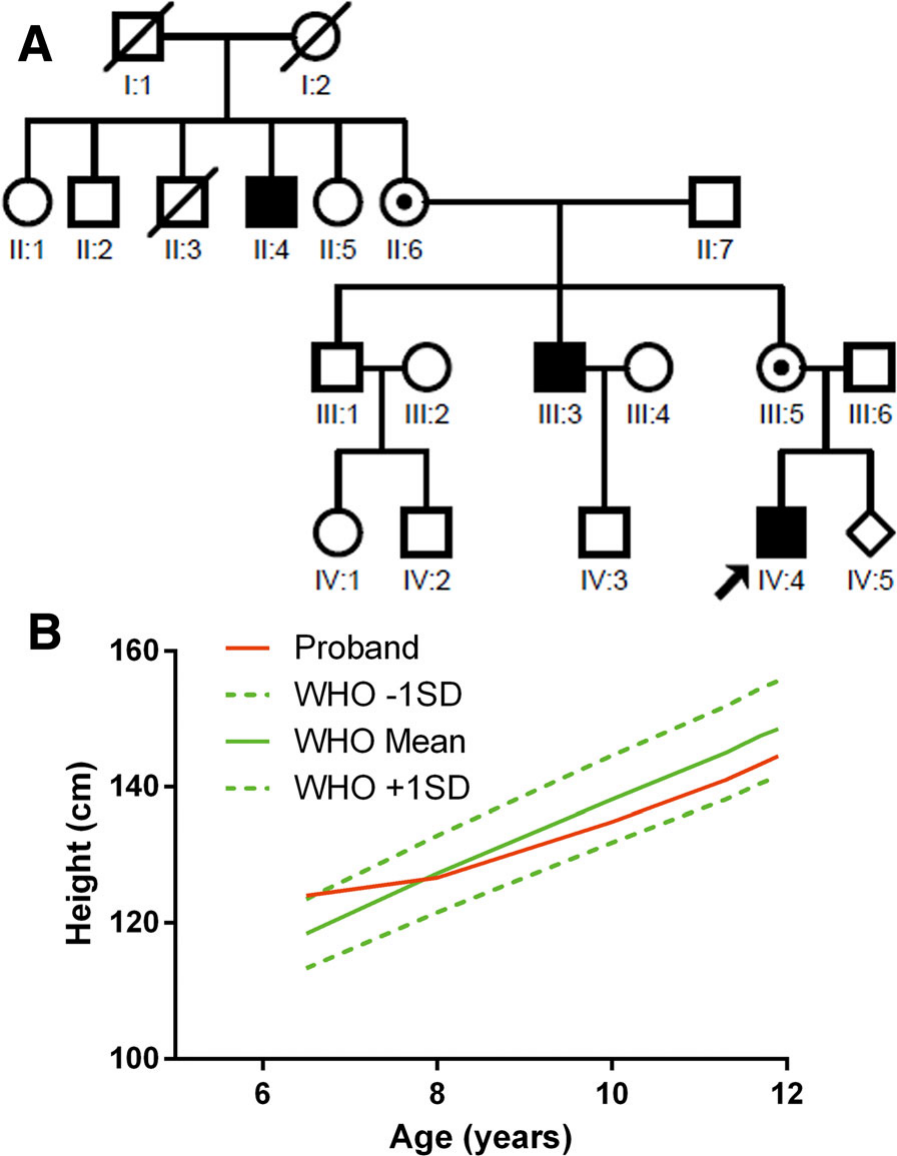
Clinical information about the Chinese NDI pedigree. **a** The pedigree of the Chinese family with nephrogenic diabetes insipidus. The affected subjects are indicated by black symbols, and the proband is indicated by an arrow; square and circle pedigree symbols indicate males and females respectively; **b** The growth curve of the proband’s height in comparison with the curves of WHO standards

The pedigree of the Chinese NDI family is shown in Fig. 1a. The proband had severe symptoms such as polyuria, polydipsia, and fatigue since infancy. He had persistent vomiting after feeding from birth to one year old. At 4 months of age he got varicella. The weight of the proband was 7 kg at 12 months. Unfortunately, NDI diagnosis was not confirmed until he was 6.5 years old, when his weight and height was 20.5 kg (about − 1 SD of WHO standards) and 1.24 m (about + 1 SD of WHO standards, Fig. 1b), respectively. The 24 h urine volume was 9 L, and specific gravity of urine was 1.003. Urine osmolality was 75 mOsmol/L and failed to rise after 24 h water deprivation (91 mOsmol/L). Diagnosis of NDI was made according to disease history and water deprivation test. His urine volume reduced 1/3 after hydrochlorothiazide treatment. However, treatment failed at the age of 13 year and his parents refused further treatment. His height growth curve was plotted in Fig. 1b. At 8–9 years, nosebleeds happened several times per month. He was very susceptible to catch a cold before the age of 10. X-ray assessment showed that his bone age was 9 years when he was 12 years old. Also, his serum uric acid level was 514 μmol/L, 25-hydroxy-vitamin D (25-OH-VD) level was 17.64 ng/ml. Serum concentration of glucose, electrolytes (Na^+^, K^+^, Ca^2+^, Cl^−^), urea nitrogen and creatinine, complete cell blood count as well as plasma osmolality were all in the reference range. Moreover, neither electrocardiogram nor brain magnetic resonance imaging showed anomaly. The two affected maternal male relatives (II:4, III:3) of the proband also had severe polyuria, polydipsia, fatigue, nocturia, failure to grow, and lower cognitive ability. All patients showed no clinical signs of immunodeficiency.

### DNA isolation and mutation detection

Genomic DNA was isolated from peripheral blood leucocytes using Tiangen Biotech (Beijing, China) Genomic DNA Purification Kit according to the manufacturer’s protocol. The entire coding sequence with the flanking intronic sequence of the AVPR2 gene was amplified using the polymerase chain reaction (PCR). To identify the deleted region around *AVPR2* gene, primer pairs were designed at 2.5-kb intervals using Genetool software and listed in Table 1. PCR products were analyzed by electrophoresis in 1.5% agarose gel. Long range PCR was done with KOD FX Polymerase Kit (TOYOBO). Finally, a ~ 2.5 kb mutant genomic fragment was amplified using forward primer P3 and reverse primer P9 and sequenced for further analysis.

**Table 1:** Primer sequence in this study

Genes	Oligonucleotide	Sequence
*AVPR2*-E1	Upper primer	5′ gggggatcctgggttctgtgc 3’
	Lower primer	5′ cccaggctcatgcagtccagaag 3’
*AVPR2*-E2A	Upper primer	5′ ctgcatgagcctggggtgtgtatc 3’
	Lower primer	5′ cgcaaagcaggcccagcagtc 3’
*AVPR2*-E2B	Upper primer	5′ accgccaccgtgccatctg 3’
	Lower primer	5′ ggccagcaacatgagtagcacaaag 3’
*AVPR2*-E3	Upper primer	5′ tggccaagactgtgaggatgac 3’
	Lower primer	5′ cccctcctacacccagctcag 3’
P1	Upper primer	5′ gggcccttcctccagattcttc 3’
	Lower primer	5′ gggcgaggaatccatgctaacc 3’
P2	Upper primer	5′ ctgccacacacccactctcac 3’
	Lower primer	5′ tggcagatgaggacgtgacag 3’
P3	Upper primer	5′ tccccaaaccaaagatattacag 3’
	Lower primer	5′ cggggtttcttcatgttgg 3’
P4	Upper primer	5′ cacgcataaccacatcactgaa 3’
	Lower primer	5′ gggcgagatattgagagcttc 3’
P5	Upper primer	5′ cccaaacagcccactaacagcaact 3’
	Lower primer	5′ cggggggtagaaggagggtgag 3’
P6	Upper primer	5′ cccgcactgtaggattccactc 3’
	Lower primer	5′ ggattgcaggtgtgagccagtc 3’
P7	Upper primer	5′ ggcgcagaggagaaggttgac 3’
	Lower primer	5′ cgcttccctgcatcttgttctc 3’
P8	Upper primer	5′ gcccctaggtgcgtgcttctc 3’
	Lower primer	5′ ggtggggagcaggcagagc 3’
P9	Upper primer	5′ tggcccagtttaacattttttgata 3’
	Lower primer	5′ cccggatctggactaggacatg 3’
qGAPDH	Upper primer	5′ gcgctgagtacgtcgtggagtc 3’
	Lower primer	5′ gagcctacagcagagaagcagacag 3’
qAVPR2	Upper primer	5′ gggccttctcgctccttct 3’
	Lower primer	5′ agggcaatccaggtgacatag 3’

### Quantitative PCR

Owing to the hemizygous state of the deletion in females, the qPCR analysis was performed using a FastStart Universal SYBR Green Master kit (Roche Applied Science, Germany) and on ABI 7900-HT system. An unrelated female control was also included to confirm successful amplification. The PCR mix was preheated at 94 °C for 5 min and then amplified in 40 cycles of 94 °C for 20 s and 60 °C for 15 s. Each PCR reaction was run in triplicate. To verify the specificity of PCR products, melting curve analysis was performed at the end of each PCR reaction. *GAPDH* was used as internal control. The sequences of all primers used are listed in Table 1. Data analysis was performed using the 2^-△△Ct^ method as previously described [13].

### Statistical analysis

Relative DNA level was expressed by mean. DNA level between patients was compared by Cruskal-Wallis H test. All statistical analyses were performed using IBM SPSS Statistics 22.0 software, and *P* < 0.05 was considered statistically significant.

## Results

**Fig. 2:**
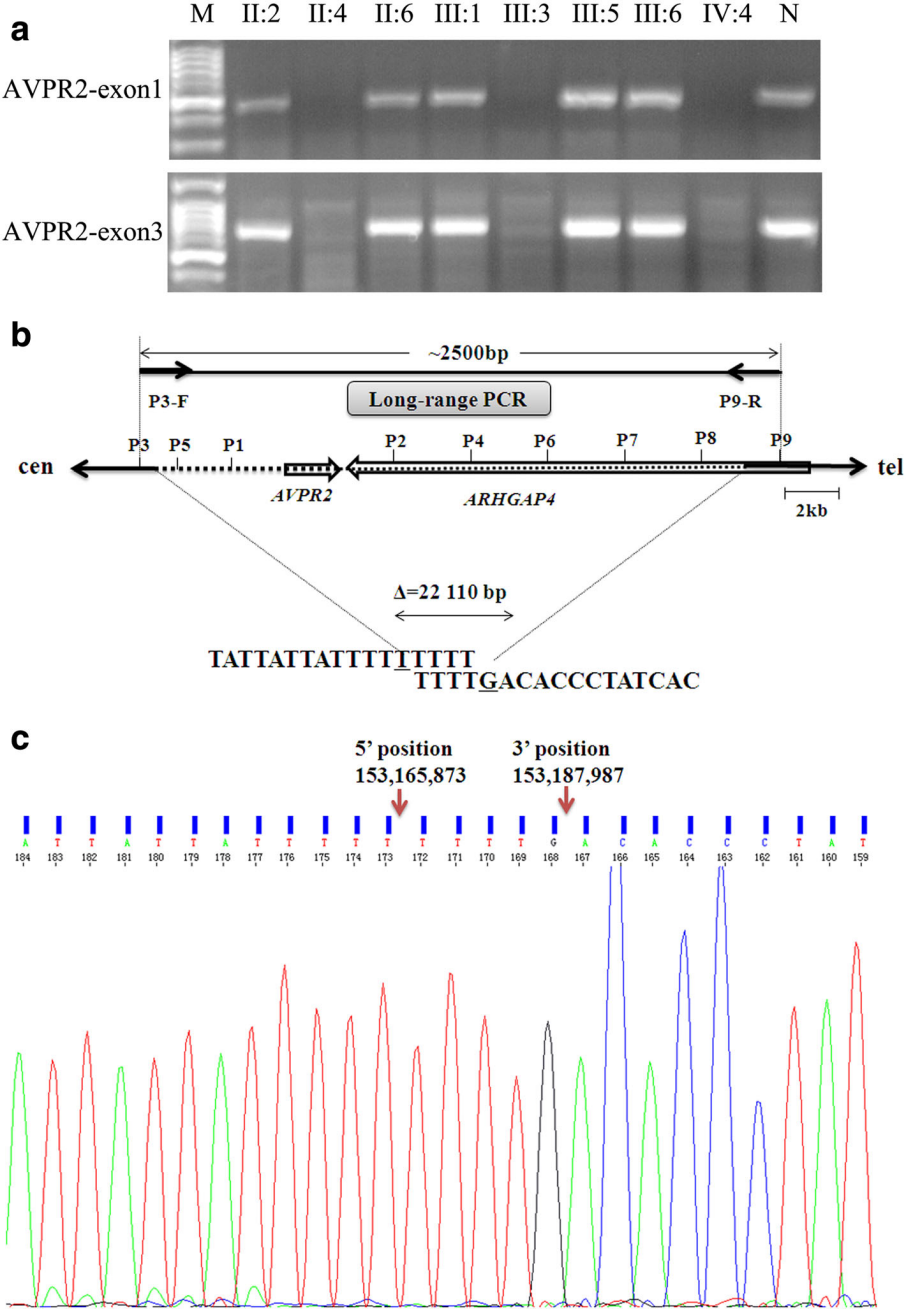
The large deletion of *AVPR2* gene in the Chinese NDI pedigree. **a** The presentation of the deletion on agarose gel electrophoresis of the NDI family members. M, 50 bp marker; N, negative control. **b** Schematic presentation of the large deletion in the Chinese NDI family. Arrows indicate genes; Solid horizontal lines indicate retained regions; Broken horizontal lines indicate deleted regions; Multiple primers were designed to be spaced every ~ 2.5 kb around the *AVPR2* gene. Long-range PCR with the forward primer of the P3 and the reverse primer of the P9 generates a PCR product of ~ 2.5 kb. Nucleotide sequences at the deletion end point, and the lowercase letters indicate deleted sequences. **c** Sequencing of deletion breakpoint in the Chinese NDI family. The possible breakpoints are indicated with arrows

According to the clinical manifestation and family history of the proband, we highly suspected the boy suffer from an X-linked NDI. Thus, we first carried out PCR to amplify and examine the exons of *AVPR2* gene. However, none of the exons was amplified. Similarly, the two affected males of his maternal relatives (II:4 & III:3) showed no PCR product from either exon 1 or exon 3 in *AVPR2* (Fig. 2a). These data strongly suggested a fragmental deletion covering *AVPR2* gene. To narrowly mapping the deletion region, we performed long-range PCR at 2.5-kb consecutive intervals to amplify the upstream and downstream of *AVPR2* gene of the proband. A total of nine pairs of primers (Table 1) were used and amplified region were schemed in Fig. 2b. After PCRs using different primer combinations, a product of ~ 2.5 kb was generated using the forward primer P3 and reverse primer P9. Subsequent sequencing and alignment revealed a deletion of 22,110 bp (chrX: 153,165,873–153,187,987; according to Human Feb. 2009 < GRCh37/hg19 > Assembly on UCSC Genome Browser). There were identical TTTT sequences between the two deletion ends (Fig. 2c). This indicates that the deletion may occur via the microhomology-mediated repair mechanism, consistent with findings in similar cases [8, 14]. The 5′ breakpoint was located within the intergenic region between *L1CAM* and *AVPR2* genes, while the 3’breakpoint was located within the first intron of *ARHGAP4* (NM_001164741). The deleted region contained the entire *AVPR2* gene and all exons of the *ARHGAP4* gene except exon 1 (Fig. 2b).

**Fig. 3:**
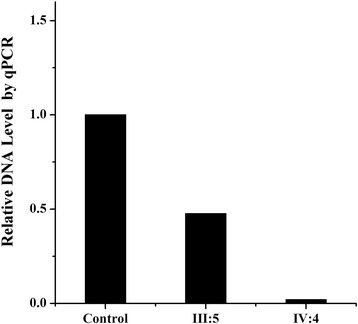
Carrier status detection of the 22-kb deletion using PCR analyses. The DNA samples were prepared from peripheral blood leucocytes. The extracted genomic DNA was spectrophotometrically quantified and diluted to 50 ng/μl. *GAPDH* was used as a control for normalization. Compared with unrelated female control, in III:5 DNA level of AVPR2 gene was decreased by half, while no PCR product was generated in IV:4 (*P* = 0.027)

Owing to the hemizygous state of the deletion in females, the qPCR analysis was performed to determine the carrier status in mother of the proband. Compared with unrelated female control, the proband’s mother showed a 50%-reduction in the copy number of *AVPR2* gene, while the proband showed no amplification of *AVPR2* (*P* = 0.027, Fig. 3).

## Discussion

In the diagnosis of NDI, mutation screening for affected children by DNA sequencing has been accepted as a standard method. In this study, we identified a novel 22.1-kb deletion covering entire *AVPR2* gene and most exons of *ARHGAP4* gene in a Chinese family with NDI. All patients in this family had remarkably developmental retardation, including short stature and intellectual disability. It is particularly noteworthy that X-ray bone age was smaller than the actual age of the proband. To our knowledge, short stature is one common feature in some severe NDI cases, compared with twelve other NDI cases previously reported [3, 5–11, 15]. It is consistent with the hypothesis that *AVPR2* is expressed in osteoblasts and osteoclasts and loss of *AVPR2* function would affect bone remodeling [16]. The serum 25-hydroxy-vitamin D level in the proband was lower than normal. Vitamin D is a bone remodeling agent. Previous studies have shown that it plays a significant role in bone formation as well as bone resorption via direct regulation of gene expression [17, 18]. These data indicate that *AVPR2* dysfunction in NDI patients may lead to the growth disturbance by both vitamin D-dependent and -independent pathways. However, its detailed mechanisms need to be further investigated.

In this study, we identified a 4 bp TTTT of identical sequence at both ends of the breakpoints using long range PCR. This indicates that deletion of *AVPR2* may occur via the microhomology-mediated repair mechanism, as indicated by findings in previous studies [8, 14, 18]. The 3′ breakpoint of the 22.1 kb deletion was located with putative long range cis-regulatory elements in the *ARHGAP4* introns [8]. All patients with the 22.1 kb deletion have the typical clinical features of cognitive impairment. This is consistent with the suggestion that these cis-regulatory elements within *ARHGAP4* introns play a role in normal cognitive function [8]. The *ARHGAP4* locus is highly conserved among species. It has been proposed that multiple long-range cis-regulatory elements in this region could function at long distances and regulate adjacent genes such as *MECP2*, *SLC6A8* or *L1CAM* [19]. However, more studies will be needed to investigate how these cis-regulatory elements influence the normal cognitive function.

There are several limitations in our study. First, due to unavailability of previous clinical records, the developmental data of other patients from the NDI pedigree could not described in this study. Second, the impact of the gross deletion on cell functions of renal tubular epithelial cell was not examined. Therefore, further experimental studies on the biological significance of *AVPR2* or *ARHGAP4* deletion are needed.

## Conclusions

In conclusion, this study identified a novel 22.1-kb deletion that is associated with polyuria, polydipsia, short stature and lower cognitive ability. Then the proband’s mother was confirmed as a carrier of the deletion by qPCR analysis. Further studies are necessary to elucidate the role of *AVPR2* gene in short stature. The results provide insights into the molecular pathogenic mechanism of NDI. Genetic analysis of the *AVPR2* gene for affected children and 3rd-generation in vitro fertilization can prevent the birth of affected children.

## Abbreviations

ARHGAP4: Rho GTPase activating protein 4
AVP: Arginine vasopressin
AVPR2: Arginine vasopressin V2 receptor
GAPDH: Glyceraldehyde-3-phosphate dehydrogenase
L1CAM: L1 cell adhesion molecule
NDI: Nephrogenic diabetes insipidus
WHO: World Health Organization
